# Multiple response optimization of the coagulation process for upgrading the quality of effluent from municipal wastewater treatment plant

**DOI:** 10.1038/srep26115

**Published:** 2016-05-18

**Authors:** Na Li, Yi Hu, Yong-Ze Lu, Raymond J. Zeng, Guo-Ping Sheng

**Affiliations:** 1 CAS Key Laboratory for Urban Pollutant Conversion, Department of Chemistry, University of Science and Technology of China, Hefei 230026, China

## Abstract

To meet the high quality standard of receiving water, the coagulation process using polyferric chloride (PFC) was used to further improve the water quality of effluent from wastewater treatment plants. Uniform design (UD) coupled with response surface methodology (RSM) was adopted to assess the effects of the main influence factors: coagulant dosage, pH and basicity, on the removal of total organic carbon (TOC), NH_4_^+^-N and PO_4_^3−^-P. A desirability function approach was used to effectively optimize the coagulation process for the comprehensive removal of TOC, NH_4_^+^-N and PO_4_^3−^-P to upgrade the effluent quality in practical application. The optimized operating conditions were: dosage 28 mg/L, pH 8.5 and basicity 0.001. The corresponding removal efficiencies for TOC, NH_4_^+^-N and PO_4_^3−^-P were 77.2%, 94.6% and 20.8%, respectively. More importantly, the effluent quality could upgrade to surface water Class V of China through coagulation under optimal region. In addition, grey relational analysis (GRA) prioritized these three factors as: pH > basicity > dosage (for TOC), basicity > dosage > pH (for NH_4_^+^-N), pH > dosage > basicity (for PO_4_^3−^-P), which would help identify the most important factor to control the treatment efficiency of various effluent quality indexes by PFC coagulation.

Biological treatment is the main process for municipal wastewater treatment. The disposal of the effluent carrying organic matters and nutrients (nitrogen and phosphorus) to the natural water bodies, will deteriorate their water quality^1^. Eutrophication and other adverse effects have been identified as growing problems in estuaries and coastal areas, and the presence of nutrients especially nitrogen (in the form of nitrate, nitrite, ammonia/ammonium) and phosphorus in wastewater leads to eutrophication^2^. With the intent to protect receiving water, regulations are becoming stringent concerning organic carbon and nutrient levels for the effluent of wastewater treatment plants (WWTPs). A clear example in China is the stringent water quality criteria for COD and nutrients (GB 18918-2002) established by China Environmental Protection Agency in 2002^3^. However, it is still difficult to meet the requirements of urban water environment quality, thus upgrading the quality of urban sewage treatment plant effluent is significant. So an efficient and economical tertiary treatment must be used to upgrade the quality of secondary effluent to surface water class V or class IV standard of China (GB3838-2002, shown in Table S1) to reduce the content of nutrients in receiving water.

Coagulation is a simple and efficient method for water and wastewater treatment, and has been widely used for drinking water, and wastewater treatment^4^,^5^. Inorganic salts of iron and aluminum are predominantly used in such water as coagulants. Recently, more researchers have focused on polyferric chloride (PFC)^6^,^7^, one novel coagulant containing a series of high positively preformed Fe(III) hydrolysis species. Compared with traditional Fe-based inorganic coagulants, PFC has both strong charge neutralization and bridging abilities, which is more effective in many cases for water treatment^8^,^9^. The efficiency of the coagulation process is governed by the various factors, such as the type, dosage and basicity of the coagulant^10^,^11^,^12^,^13^,^14^,^15^, pH in the solution^16^,^17^,^18^, mixing speed and time^19^,^20^ etc. Thus, before application of PFC for the effluent treatment, a proper optimization of these factors is needed to increase the treatment efficiency. In this regard, the conventional method named one-variable-at-a-time may be used to obtain the optimal conditions, but it fails to resolve the relationship between multi-variables due to the complex influences of these factors.

Response surface methodology (RSM) is an effective and efficient mathematical statistics method to build models, evaluate the effects of multiple variables and determine the optimal conditions to give desirable responses, which overcomes the limitation of the conventional method successfully^21^,^22^,^23^,^24^. In general, for the experimental design methods used in RSM, such as central composite design, if the numbers of experimental factors are increased, the numbers of coefficient of the quadratic model equations and trials are increased exponentially. In order to overcome this disadvantage, uniform design (UD) was proposed by Fang (1978). The numbers of the experimental trails using UD only are determined by the level of the factors, not by the number of the factors. Compared to the traditional experimental design method, UD is able to select experimental points uniformly in the experimental region, in which the selection of representative points in experimental domain depends on “uniformly dispersed”, regardless of the “neat comparable”. It provides no strong assumptions on the model and can be used even though the basic model between the response and the factor is unknown or partially unknown, and it can accommodate the number of levels for each factor in the experimental design^25^. Thus, UD coupled with RSM will contribute to accomplish the optimization of a complex multi-factors process with the fewest multi-levels experimental trials.

The objective of this study was to develop a multi-responses optimization method by an integrated UD-RSM experimental design for the coagulation process to improve the effluent quality from a WWTP. For this purpose, the coagulant PFC was used and the coagulation parameters in terms of coagulant dosage, pH and basicity were optimized via an integrated UD-RSM experimental design and a multi-responses optimization approach. The quality of effluent after the optimization of the coagulation process was evaluated in terms of the level of surface water class V standard of China. It is anticipated that the novel optimization strategy used in this study may provide a useful approach for other complicated systems in the environmental field.

## Results and Discussion

### Optimization for TOC removal

TOC removal is an important indicator for the treatment efficiency of the coagulation process. As listed in Table 1, the different coagulation conditions had distinct difference in the residual TOC concentration of the effluent. The following equation was obtained by modeling the experimental results with the nonlinear regression method:


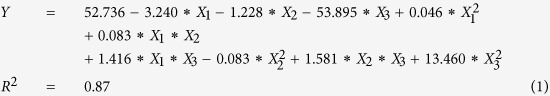


**Figure 1:**
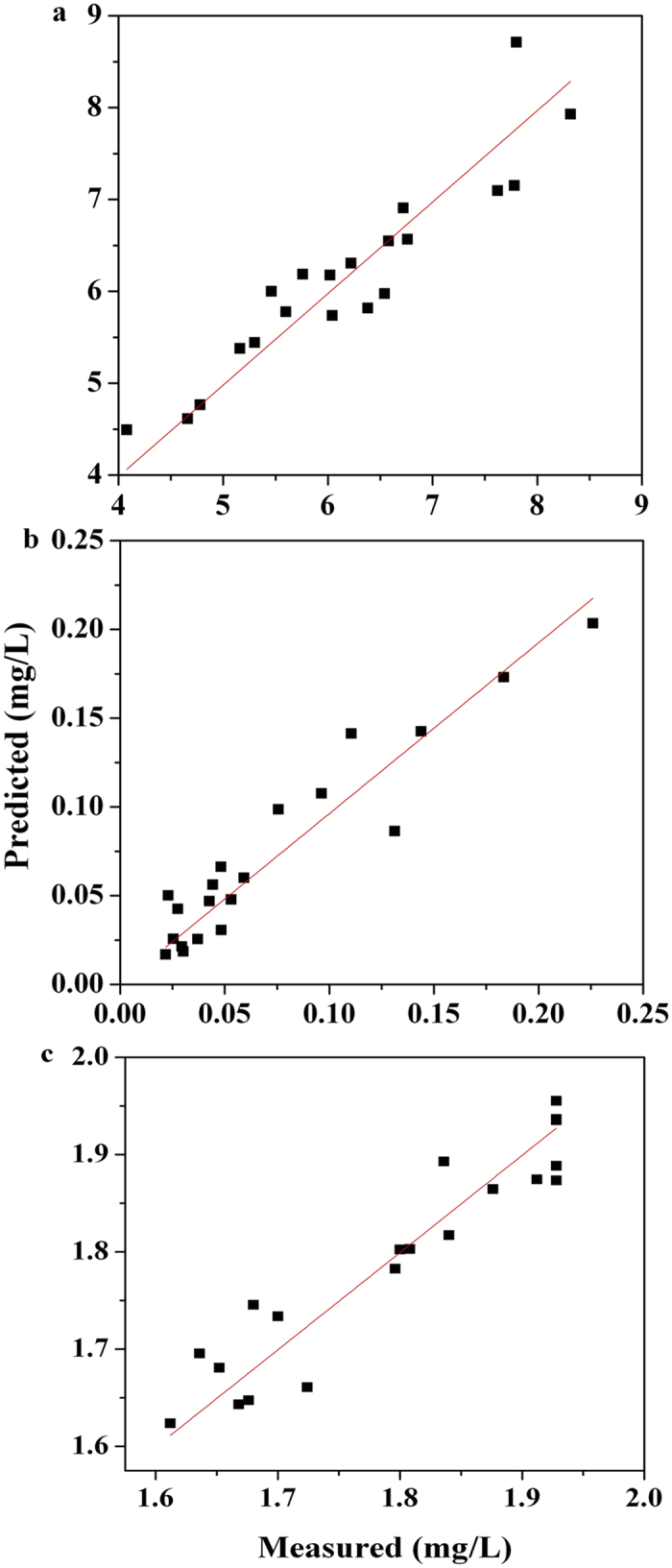
Relationship between the predicted and measured (**a**) residual TOC; (**b**) residual PO_4_^3−^-P; and (**c**) residual NH_4_^+^-N.

Statistical testing of the model was executed with the Fisher’s statistical test for analysis of variance (ANOVA). The quadratic regression indicates that the model was significant, because the value of F-statistic (the ratio of mean square ascribed to regression to the mean square to the real error) of 14.644 was much greater than F_0.05,1,15_ (4.543). The high value of coefficient of determination (R^2^ = 0.87) and the low P-values (0.05) verified the sufficiency of the model. The plots of the predicted TOC residual concentration versus the measured ones are shown in Fig. 1a. Most points distributed near to the straight line, indicating that the regression model was able to predict these residual TOC concentrations. The optimized conditions to obtain the lowest TOC residue concentration were obtained by setting the partial differential of the response (Eq. 1) to zero as follows: coagulant dosage of 24 mg/L, pH of 7.5 and basicity of 0.30, where the minimum concentration of TOC was calculated to be 1.17 mg C/L, which was equivalent to 4.7 mg COD/L (according to the empirical value obtained through analyzing the relationship between TOC and COD in sewage treatment plants^26^). The removal efficiency was 85.9%.

**Figure 2:**
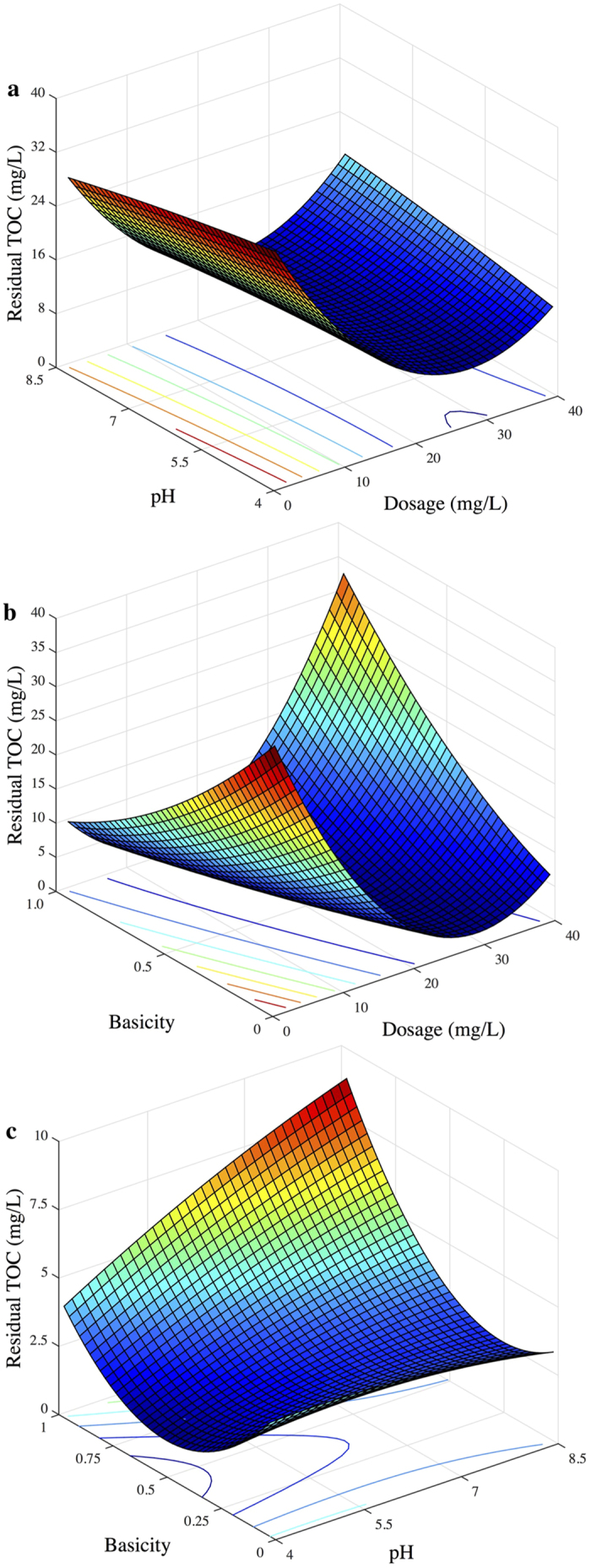
3D surface graphs and contour plots of the residual TOC concentration: effect of variables (**a**) X_1_-X_2_; (**b**) X_1_-X_3_; and X_2_-X_3_.

The effects of coagulant dosage, pH and basicity on the TOC concentration as the response are shown in the 3-dimentional response surface graph (Fig. 2). The distinct peak in the response surfaces indicates that the optimal conditions were exactly in the experimental region. In other words, there were significant interactive effects on TOC removal between the three factors.

### Optimization for PO_4_^3−^-P removal

PO_4_^3−^-P content is regarded as one of the important factors controlling water eutrophication^27^. It is essential to remove PO_4_^3−^-P from the effluent before it is discharged to receiving water. The effects of various coagulation operation parameters on the removal of PO_4_^3−^-P using PFC could be described by the following equation, which was obtained by modeling the results in coagulation experiments with the nonlinear regression method:


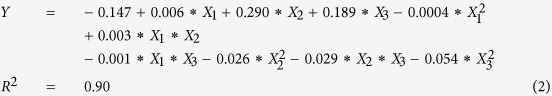


The results of F = 18.175 > F _0.05,1,15_ = 4.543 and R^2^ = 0.90 for the PO_4_^3−^-P removal efficiency illustrated that the second-order polynomial model fitted the experimental results well. Figure 1b shows that the measured versus predicted plot values were distributed evenly near to the straight line. From Eq. 2, the optimized conditions for the minimum residual PO_4_^3−^-P concentration were calculated to be: coagulant dosage of 32 mg/L, pH of 7.0 and basicity of 0.25. Under these conditions the residual PO_4_^3−^-P concentration was calculated to be 0.01 mg/L, and the removal efficiency was 99.9%.

**Figure 3:**
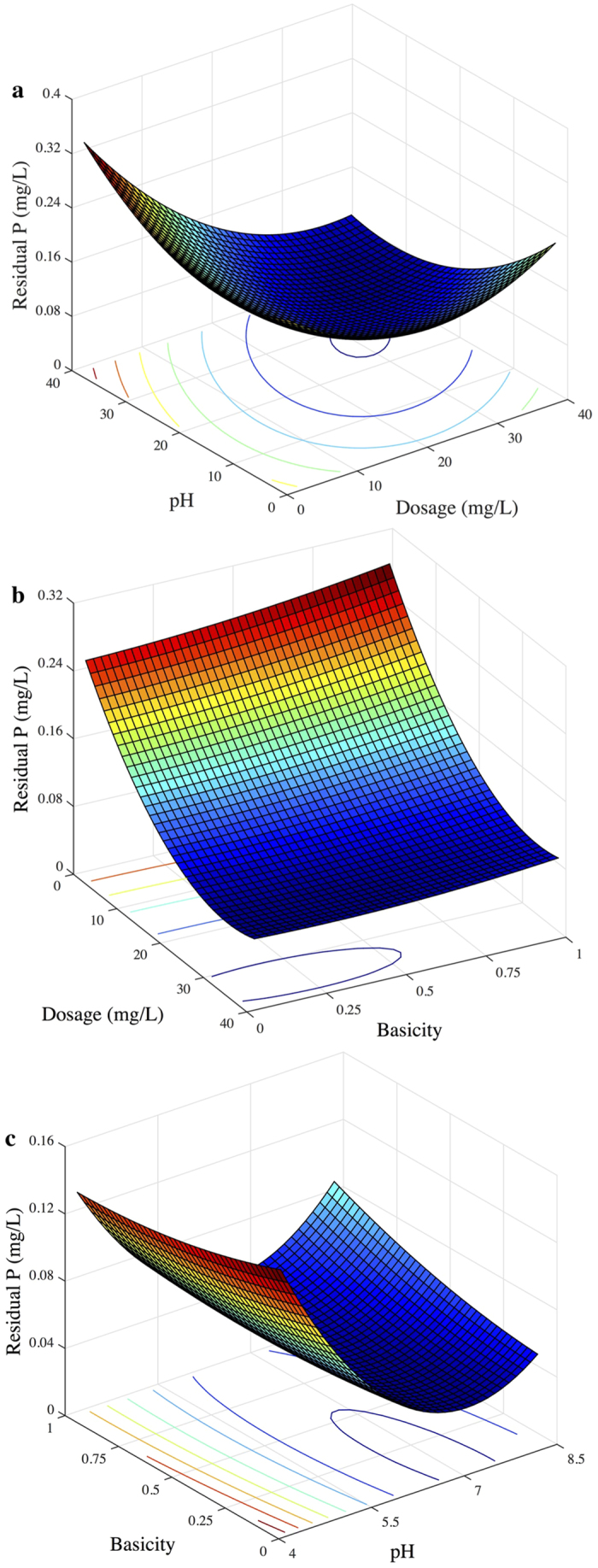
3D surface graphs and contour plots of the residual PO_4_^3−^-P concentration: effect of variables (**a**) X_1_-X_2_; (**b**) X_1_-X_3_; and X_2_-X_3_.

When the residual concentration of PO_4_^3−^-P was chosen as the response, the response surfaces of the quadratic model that one variable kept at the optimal value and the other two varied within the experimental ranges, respectively, are illustrated in Fig. 3. The distinct peak in the response surfaces indicates that the optimal conditions were exactly located inside the design boundary. The elliptical contour plots imply that there were significant interactive effects on PO_4_^3−^-P removal between the three factors.

### Optimization for NH_4_^+^-N removal

The regression model to describe the NH_4_^+^-N removal efficiency of the coagulation experiments was obtained using the backward regression method:


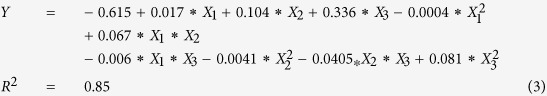


The quadratic regression indicates a high significance of the model, because the value of F-statistic of 6.30 was much greater than F _0.05,1,14_ (4.60). The high value of the correlation coefficient (R^2^ = 0.85) suggests a good agreement between the measured and predicted values of the residual NH_4_^+^-N concentration (Fig. 1c). Then, the optimal conditions for maximum NH_4_^+^-N removal were calculated to be: coagulant dosage of 27 mg/L, pH of 8.5 and basicity of 0.001. In this case, the minimum concentration of residual NH_4_^+^-N was calculated to be 1.52 mg/L and the removal efficiency was only 20.6%.

**Figure 4:**
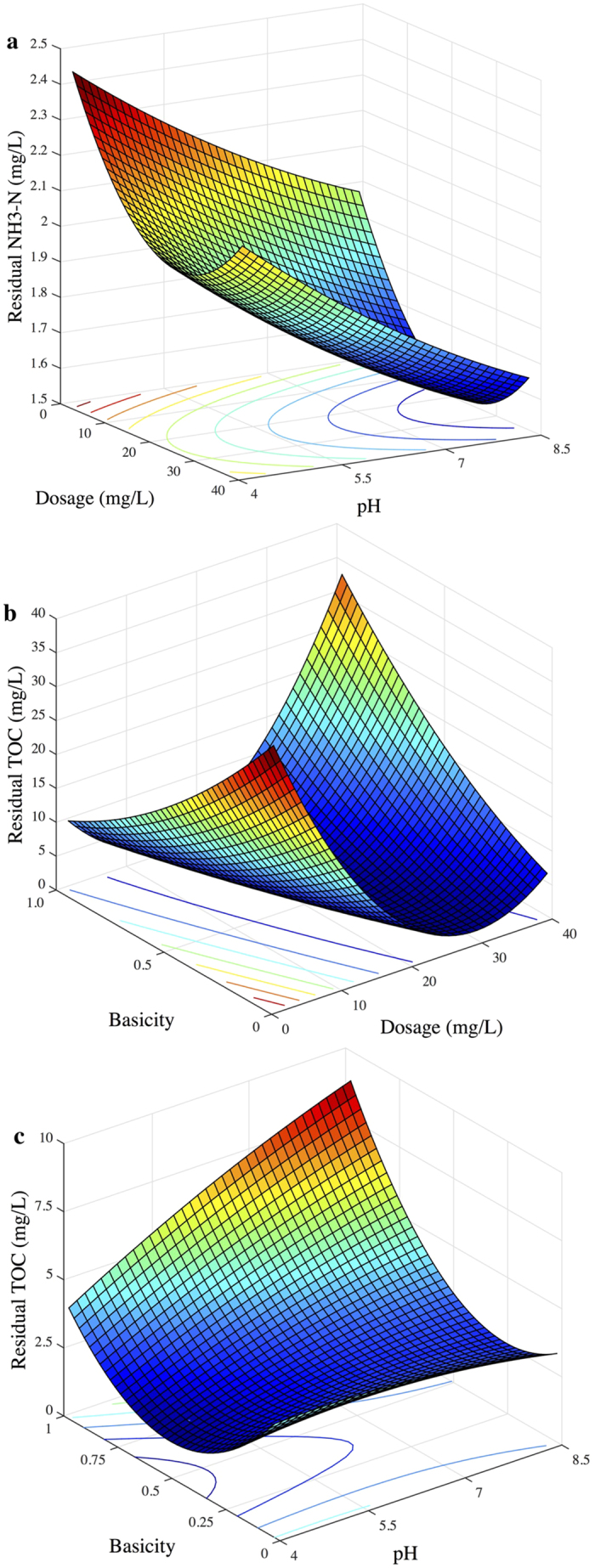
3D surface graphs and contour plots of the residual NH_4_^+^-N concentration: effect of variables (**a**) X_1_-X_2_; (**b**) X_1_-X_3_; and X_2_-X_3_.

The response surfaces of the quadratic model with the concentration of residual NH_4_^+^-N as the response are shown in Fig. 4. The contour plots imply that there were significant interactive effects on NH_4_^+^-N removal between dosage and pH, dosage and basicity, as well as pH and basicity. This was evidenced by the obvious peak in the response surface, in which the optimal conditions were exactly located in the experimental range.

### Removal mechanisms for TOC, PO_4_^3−^-P and NH_4_^+^-N by PFC

The residual organics in the effluent from the biological WWTPs include humic substances, proteins, microbial products and other inert matters, most of them are biopolymers and in the form of colloids^28^,^29^. The removal mechanisms of these organics through coagulation include a combination of charge neutralization, entrapment, adsorption and complexation with the iron based coagulant into insoluble particulate aggregates^4^. PFC was pre-hydrolyzed in the preparation process, and when it was dosed to the solution, it could slow down the rate of hydrolysis of PFC species through dilution. So PFC species might have sufficient time to react with the organic matters. More polymeric species with higher positive charge density were formed with the increase of basicity, which benefited TOC removal by adsorption and charge neutralization. Under these conditions, the coagulant PFC exhibited good charge neutralization, bridging and sweep-floc abilities, which contributed the high TOC removal efficiency.

The coagulation of PO_4_^3−^-P with Fe(III) salts includes two major mechanisms: formation of Fe-hydroxo-phosphate complexes, Fe(OH)_3−x_(PO_4_)_x_, which either absorbs onto positively charged Fe(III) hydrolyzed species or precipitates with Fe(III) hydrolyzed products as the centers; and adsorption of PO_4_^3−^-P ions with polymeric species^9^,^30^. The pH plays an important role in the PFC coagulation process, and the PFC flocs formed at a neutral condition would give the largest floc size^7^. In addition, basicity is one of the important factors that affects the nature of Fe(III) species, thereby affecting coagulation performance. Therefore, under the optimized conditions, the coagulant PFC exhibited good absorption and sweep-floc abilities, and had a good performance for PO_4_^3−^-P removal.

Because the coagulation process does not remove NH_4_^+^-N directly, the removal efficiency of NH_4_^+^-N was not very high. After PFC was dosed to the solution, the pre-hydrolyzed Fe species would enmesh and co-precipitate with the colloidal particles in wastewater, and then settle together. Partial of NH_4_^+^-N could bind to the surface of the negatively charged colloidal particles by electrostatic attraction^31^. As a result, partial NH_4_^+^-N could be removed from the wastewater together with the colloid particles.

Different removal mechanisms lead to the different optimal removal conditions, which are consistent with the above results. Furthermore, to confirm the validity of the statistical experimental strategies, additional confirmation experiments based on the optimized coagulation conditions above were conducted. The chosen conditions and results are listed in Table 2. The measured concentrations of residual TOC, PO_4_^3−^-P and NH_4_^+^-N were close to the calculated values by their respective regression models. This result demonstrated that UD-RSM approach was useful for optimizing the coagulation process parameters, in terms of minimizing the effluent concentrations of TOC, PO_4_^3−^-P and NH_4_^+^-N.

### Optimization for multiple responses of TOC, NH_4_^+^-N and PO_4_^3−^-P removal

The optimization for individual removal of TOC, PO_4_^3−^-P or NH_4_^+^-N achieved under the different optimal conditions is a big concern in real wastewater treatment. Thus, it is necessary to consider the comprehensive removal of TOC, PO_4_^3−^-P, and NH_4_^+^-N in practical application. In this work, a desirability function approach^32^,^33^ was used to optimize multiple responses in order to obtain a good quality of the effluent to match high water quality of receiving water. The completely desirable values of the response residual concentrations of TOC, PO_4_^3−^-P and NH_4_^+^-N were set at 1.19, 0.01 and 1.52 mg/L, respectively, which were the lowest residual concentrations of TOC, PO_4_^3−^-P, and NH_4_^+^-N obtained under the individual optimal conditions. And the completely undesirable values of those responses were above 10 (equivalent to 40 mg COD/L), 0.4 and 2.0 mg/L, respectively, which was based on surface water class V of China^3^. Then the values of desirability function *D* obtained by calculating the geometric mean of the three individual ideal functions at various experimental conditions are listed in Table 1, and was regressed using the following equation:


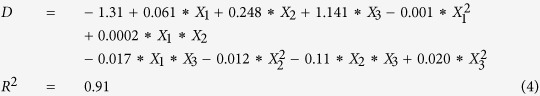


**Figure 5:**
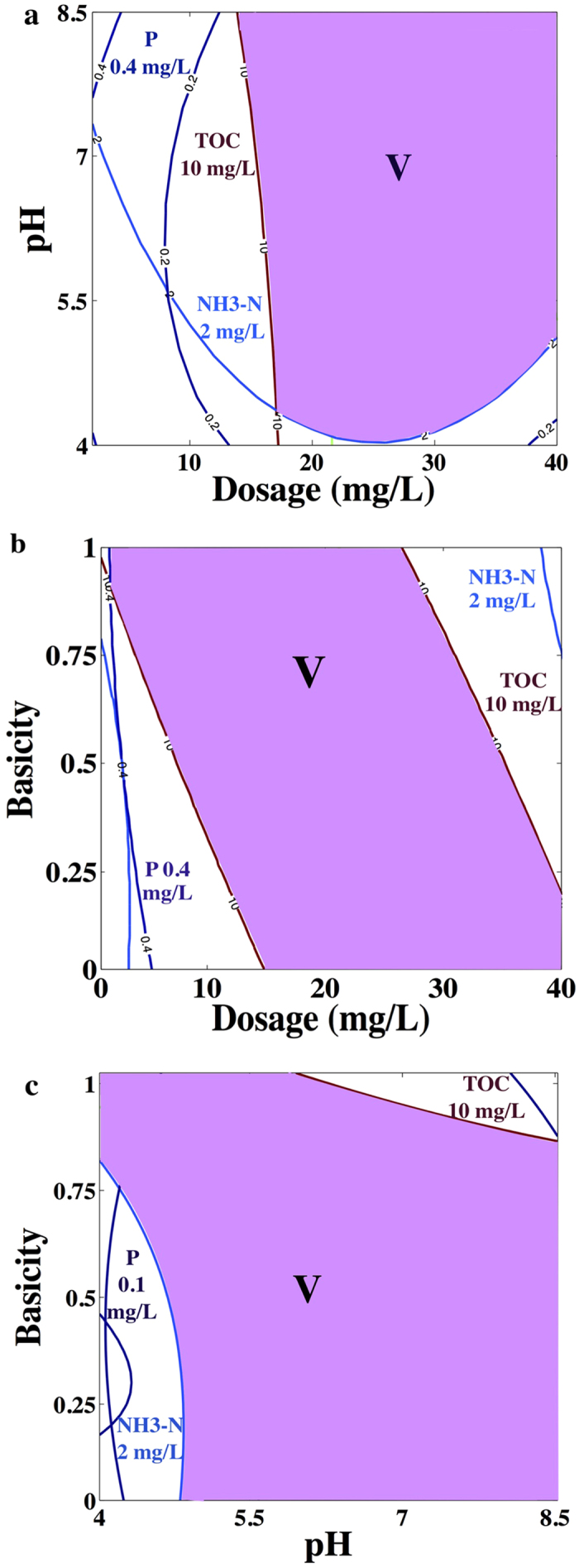
Overlay plot for the optimal region (surface water class V standard of China).

The optimal conditions were calculated to be: dosage of 28 mg/L, pH 8.5, and basicity 0.001, respectively. The corresponding residual concentrations of TOC, PO_4_^3−^-P, NH_4_^+^-N concentration and D value were 1.41 mg/L, 0.03 mg/L, and 1.53 mg/L and 0.90, respectively. The overlay plot for the optimal region is presented in Fig. 5. The shaded portion gave the permissible values of the variables by defining the desired limits of the concentrations of TOC, PO_4_^3−^-P and NH_4_^+^-N as 10 mg/L, 0.4 mg/L and 2.0 mg/L, respectively, and the treated effluent satisfied surface water Class V standard of China.

A confirmation experiment under the compromised conditions was carried out in duplicates, and the concentrations of TOC, PO_4_^3−^-P, and NH_4_^+^-N in the obtained effluent were 2.49 (equivalent to 10.0 mg COD/L), 0.05, and 1.52 mg/L, respectively, and these results were in good agreement with the predicted ones, and the differences between predicted and experimental values were 0.90, 0.02, and 0.01 mg/L, respectively. As shown in Table 3, the effluent index, including the concentrations of TOC, PO_4_^3−^-P, and NH_4_^+^-N, dropped significantly after the treatment under the optimal conditions, especially for the PO_4_^3−^-P concentration (with a removal efficiency of 90.9%), which was reduced one order of magnitude compared to the value before the treatment, and the treated effluent could reach to surface water Class V standard of China.

### Influential priority of the three factors

GRA method was used to evaluate the influential degrees of dosage, pH, and basicity on the removal of TOC, NH_4_^+^-N and PO_4_^3−^-P by coagulation using PFC. The grey relational grades γ of these factors for the treatment performance were calculated to be:


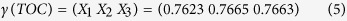



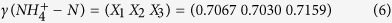



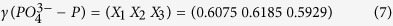


Results showed that the influential priorities on the removal of TOC, NH_4_^+^-N and PO_4_^3−^-P in the effluent after coagulation were quite different. The pH showed the most significant effect on TOC removal, followed by basicity. Dosage exhibited a less-significant effect. For NH_4_^+^-N, the influential priorities were in the order of: basicity > dosage > pH. In contrast, the influential priorities of these factors on PO_4_^3−^-P removal were in the order of: pH > dosage > basicity. The results in this work also demonstrated that pH was the most significant factor affecting the removal of organic matters and PO_4_^3−^-P from the WWTP effluent by coagulation using PFC. The pH determines the hydrolysis of PFC species and floc sizes, which in turn affect the coagulation mechanism. Because of the slow hydrolysis of PFC under acidic condition, the charge neutralization dominates the coagulation process of PFC. The hydrolysis tendency of PFC becomes much larger with pH increased, while bridging, absorption and sweep-floc play important roles in coagulation process of PFC. Furthermore, coagulants give bigger final floc sizes at neutral and alkaline conditions than acidic conditions which benefit the removal for PO_4_^3−^-P. This result agreed with previous studies, in which pH was also found to play an important role in the coagulation process using PFC^6^,^7^. In contrast, basicity, rather than pH and dosage that are usually considered in the coagulation process for wastewater^34^ or drink water^35^ treatment, was found to be the crucial factor influencing the removal of NH_4_^+^-N in this study. Basicity determines the species of pre-hydrolyzed products in preparation process of PFC. The hydrolyzed Fe species, such as amorphous ferric hydroxide precipitates, exhibit good enmesh and co-precipitate abilities with the colloidal particles, and in turn cause the reduction of NH_4_^+^-N bound to them.

### Implications of this study

An integration of UD and RSM, with a rational statistical basis, is proved to be a valid approach for the optimization of the coagulation process for the treatment of the effluent from WWTP. The response variable was fitted by a second-order model when using the regression method to describe the relationship between the dependent output variable and the independent variables with the fewest multi-levels experimental trials. The value of F-statistic was much greater than F_0.05_ indicated that the model was significant, and the high value of coefficient of determination (R^2^ > 0.85), a sufficient large residual degree of freedom (17, much higher than 5) and the low P-values (0.05) verified the sufficiency of the model, these all illustrated the second-order model was suitable and effective in this study. Under the optimized conditions, the treated effluent could reach to surface water Class V standard of China. Due to serious water pollution in China, the WWTPs need to upgrade to meet the increasing strict discharge regulation, indicating that the sewage treatment plants face the problem of upgrading and reconstruction. The coagulation process is easy to implement without big changes in structures or technological process of sewage treatment plant. This integrated optimized approach for PFC coagulation also could further improve the effluent quality to meet the surface water Class V standard of China, which may significantly reduce the pollution of the receiving water body. However, the efficiency of the coagulation process is governed by the various factors, such as the type, dosage and basicity of the coagulant, pH in the solution, the characteristics of wastewater, e.g. anion, cation, the type and concentration of pollutants etc. Thus, the optimized parameters for coagulation obtained in this study may not be applicable for other sewage treatment plants, but it is suitable to upgrade the quality of the effluent from sewage treatment plants through coagulation by the multi-response optimization method.

Meanwhile, this study shows that this UD approach effectively optimizes the coagulation process by taking advantage of few data sets and factors with different levels, which is very attractive for the processes where data are obtained hardly or expensively. Thus, this optimization approach can be applicable for other complex or multivariate systems in the environmental field. UD focuses on the selected experimental points distributing uniformly within the factor space for all the three key factors with different levels influencing the efficiency of this process, i.e., dosage, pH and basicity in this study. Thus most response information is obtained through the fewest numbers of the experiments and multilevel factors. These characteristics are the advantages of UD compared with other experimental design approaches.

However, in addition to the treatment efficiency, the cost is also a big concern in practical application. GRA is adopted to assess the influential priorities of factors, which does a favor for selecting the most important factor, ultimately achieving a balance between the treatment efficiency and the cost. For example, GRA results show that basicity and pH have the most and least significant effect on the residual NH_4_^+^-N concentration, respectively. However, the pH is usually uncontrollable in WWTPs before the coagulation process, while the basicity of commonly used coagulant PFC is determined with the experience without optimization during practical application. The results of UD and RSM reveal that NH_4_^+^-N removal could be improved through adjusting the basicity of PFC. Therefore, in the practical application, the sewage of high ammonium content could be treated without pH adjustment, which could reduce the operation cost to improve the effluent quality. All of these indicate that the results of UD, RSM and GRA are complementary. Through the multi-responses optimization of coagulation (a simple and efficient water treatment process), the water quality of the effluent from a WWTP could be upgraded to surface water class V of China.

## Conclusions

In present work, a coagulation process with PFC was employed to treat the effluent from a WWTP to improve the effluent quality. A multiple responses optimized method RSM coupled with UD was used to successfully optimize the process, and the individual and interactive effects of the main influential factors were evaluated. An optimal condition of coagulant dosage 28 mg/L, pH 8.5 and basicity 0.001 was obtained when considering the removal of TOC, PO_4_^3−^-P and NH_4_^+^-N simultaneously. More importantly, the water quality of the treated effluent after PFC coagulation could reach surface water class V of China under the optimal region. This indicated that the PFC coagulation process after optimization could efficiently improve the quality of the effluent from WWTPs to meet more and more strict wastewater discharge policy.

## Methods

### PFC preparation and coagulation experiment

PFC used in the experiments was prepared by slowly adding Na_2_CO_3_ powder to a certain concentration of FeCl_3_ solutions under the stirring conditions at room temperature. The amount of Na_2_CO_3_ added to FeCl_3_ solution was varied depending on the target basicity B (B = [OH^−^]/[Fe^3+^]) at 0, 0.25, 0.50, 0.75 and 1.00. After the foam disappeared and the solution became transparent, Na_2_HPO_4_ was added to the solution as a stabilizer ([Na_2_HPO_4_]/[Fe] = 0.08). The concentration of Fe was 7% (w/w) in the PFC solution. The dosages of PFC were calculated as mg/L of Fe during coagulation. All reagents used were of analytical grade.

The raw effluent was sampled from the secondary sedimentation tank in a local municipal wastewater treatment plant in Hefei, China, and stored at 4 °C. Before each experiment, the temperature of all samples was controlled at the room temperature of 24 ± 1 °C. The raw effluent samples were characterized in terms of total organic carbon (TOC), pH, NH_4_^+^-N, PO_4_^3−^-P and NO_3_^−^-N. The properties of the samples listed in Table 4 were: TOC 10.20 ± 1.55 mg/L, PO_4_^3−^-P 0.55 ± 0.05 mg/L, NH_4_^+^-N 2.15 ± 0.50 mg/L, NO_3_^−^-N 13.75 ± 0.25 mg/L, pH 7.3 ± 0.2.

A series of jar tests were carried out in 1-L breakers. After the solution pH was adjusted from 4 to 8.5 by adding 0.1 M HCl or NaOH solutions, the PFCs with different basicity were added to the solution with a dosage of 2–40 mg/L. The samples were immediately stirred at 200 rpm for 2 min, and then stirred at a lower speed of 30 rpm for 20 min, followed by a settlement for 30 min. The samples were taken from about 2 cm below the surface and then filtered with 0.45 μm membrane to measure TOC, NH_4_^+^-N, PO_4_^3−^-P.

PO_4_^3−^-P and NH_4_^+^-N were measured by a water quality autoanalyzer (Aquakem 200, ThermoFisher, Finland) following the standard methods (APHA, 1998), and TOC was measured via a TOC analyzer (multi N/C 2100, Analytikjena, Germany).

### Experimental design with UD

U_n_(q^m^) can be used to describe UD table, wherein, U, *n*, *q* and *m* represent the UD, the number of experimental trials, the number of levels and the maximum number of factors, respectively. In this study, coagulant dosage (X_1_), pH (X_2_), and basicity (X_3_) were selected as three independent variables in the coagulation process. Twenty levels, ten levels and five levels were chosen for X_1_, X_2_, X_3_, respectively, to investigate the effect and interaction of the factors on coagulation. When considering the operability of all factors and ensuring the accuracy of the experiments, a mixed-level UD table with the different number of levels of each factor, U_20_ (20*10*5) with discrepancy of 0.1664 in uniformity, was chosen to carry out the experiments. The range and levels of each factor are listed in Table 1.

The responses included the removal of TOC, NH_4_^+^-N and PO_4_^3−^-P, which represented the overall wastewater treatment efficiency. The response variable was fitted by a second-order model using the regression method to describe the relationship between the dependent output variable and the independent variables.


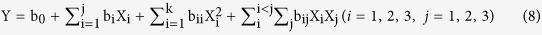


where X_i_ refers to the independent input variable that influences output variable Y; b_0_, b_i_, b_ii_ and b_ij_ are the constant regression coefficients, the *i*^th^ linear coefficient, the *i*^th^ quadratic coefficient and *ij*^th^ interactive coefficient of the equation, respectively.

Uniform Design Software 3.0 (http://www.math.hkbu.edu.hk/Uniform Design/software) was used to evaluate the parameters of the response equations, and corresponding analysis on variations were assessed using MATLAB 7.0. Meanwhile, three-dimensional response surfaces or two-dimensional contour curves were illustrated to give the visual responses of TOC, NH_4_^+^-N and PO_4_^3−^-P. The confirmation experiments under individual and integrated optimal removal conditions were conducted to testify the accuracy of statistical experimental results.

### Optimization for multiple responses

Desirability function approach is the most common method of optimizing multiple responses^32^,^33^,^36^. For each system response *Y*_*i*_, ideal function *d*_*i*_(*Y*_*i*_) is assigned the value between the ranges of 0 to 1. Specifically, *d*_*i*_(*Y*_*i*_) = 0 and 1 mean that *Y*_*i*_ is a completely undesirable value and a completely desirable response, respectively. Then the general desirable function is obtained by calculating the geometric mean of all individual desirable function together:


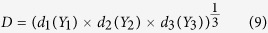


The specific calculation method for the individual desirable function value is as follows:


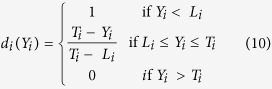


where *T*_*i*_ and *L*_*i*_ represent the completely undesirable value and completely desirable value, respectively.

### GRA

It is essential to understand the most significant influential parameter for the coagulation process. To prioritize the various factors on the treatment efficiency, grey relational analysis (GRA)^37^ was adopted to identify the interrelationships between multiple factors and variables. The grey relational grade γ is obtained by calculating the average values of all the grey relational coefficients with the methods mentioned in previous research^38^,^39^ as follows:


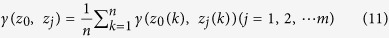


where z_0_ and z_j_ are the normalized data of the input factors and the output variables, respectively. γ(z_0_(k) and z_j_(k)) are the grey relational coefficient.

The larger grey relational grade γ indicates the influential degree of the factor on the system is greater.

## Additional Information

**How to cite this article**: Li, N. *et al*. Multiple response optimization of the coagulation process for upgrading the quality of effluent from municipal wastewater treatment plant. *Sci. Rep*. **6**, 26115; doi: 10.1038/srep26115 (2016).

## Supplementary Material

Supplementary Information

## Figures and Tables

**Table 1:** UD experimental design and the response results for coagulation processes using PFC.

Run	Factors						Responses			
	Code			Level			Residual	Residual	Residual	D Value
	X_1_	X_2_	X_3_	X_1_ (mg/L)	X_2_	X_3_	TOC (mg/L)	NH_4_^+^-N (mg/L)	PO_4_^3−^-P (mg/L)	
1	1	6	5	2	6.5	1	8.71	1.80	0.23	0.31
2	2	1	5	4	4	1	7.15	1.88	0.18	0.37
3	3	6	4	6	6.5	0.75	7.93	1.84	0.14	0.38
4	4	1	4	8	4	0.75	6.55	1.93	0.11	0.37
5	5	7	3	10	7	0.5	7.10	1.80	0.10	0.49
6	6	2	3	12	4.5	0.5	6.19	1.93	0.08	0.40
7	7	7	2	14	7	0.25	6.91	1.68	0.05	0.60
8	8	2	2	16	4.5	0.25	6.18	1.93	0.13	0.37
9	9	8	1	18	7.5	0	6.57	1.67	0.05	0.63
10	10	3	1	20	5	0	6.31	1.93	0.06	0.40
11	11	8	5	22	7.5	1	5.97	1.68	0.04	0.66
12	12	3	5	24	5	1	4.49	1.70	0.04	0.72
13	13	9	4	26	8	0.75	5.78	1.65	0.03	0.70
14	14	4	4	28	5.5	0.75	4.77	1.81	0.04	0.62
15	15	9	3	30	8	0.5	5.44	1.64	0.03	0.73
16	16	4	3	32	5.5	0.5	4.61	1.93	0.05	0.46
17	17	10	2	34	8.5	0.25	5.74	1.72	0.03	0.65
18	18	5	2	36	6	0.25	5.38	1.91	0.03	0.47
19	19	10	1	38	8.5	0	6.00	1.61	0.02	0.72
20	20	5	1	40	6	0	5.82	1.84	0.02	0.55

Note: X_1_, X_2_, and X_3_ represents to coagulant dosage, pH and basicity, respectively.

**Table 2:** Measured and calculated values for the confirmation experiments.

Run	Condition	Response values	Measured value (mg/L)	Calculated value (mg/L)
21	PFC dosage: 24.0 mg/L pH: 7.5 Basicity: 0.30	Residual TOC concentration	1.85 ± 0.15	1.17
22	PFC dosage: 32.0 mg/L pH: 7.0 Basicity: 0.25	Residual PO_4_^3−^-P concentration	0.05 ± 0.02	0.01
23	PFC dosage: 27 mg/L pH: 8.5 Basicity: 0	Residual NH_4_^+^-N concentration	1.52 ± 0.05	1.53

**Table 3:** Effluent quality from a local wastewater treatment plant before and after coagulation treatment under optimal conditions (Dosage of 28 mg/L, pH of 8.5, basicity of 0.001).

Parameters	Before treatment	After treatment (optimal)	Treatment efficiency (%)
TOC (mg/L)	8.65–11.75	2.49 ± 0.18	73.2 ± 0.2
PO_4_^3−^-P (mg/L)	0.55 ± 0.05	0.05 ± 0.01	90.9 ± 0.2
NH_4_^+^-N (mg/L)	1.65–2.61	1.52 ± 0.05	18.8 ± 0.2

**Table 4:** Characteristics of effluent from the wastewater treatment plants.

Parameters	Measured
TOC (mg/L)	8.65–11.75
PO_4_^3−^ (mg/L)	0.55 ± 0.05
NO_3_^−^ (mg/L)	13.75 ± 0.25
NH_4_^+^ (mg/L)	1.65–2.61
pH	7.30 ± 0.20
Temperature (°C)	25
