# Supplementary material for: Multiple response optimization of the coagulation process for upgrading the quality of effluent from municipal wastewater treatment plant
Source: Sci Rep. 2016 May 18;6:26115. doi: 10.1038/srep26115 (PMC4870683; doi:10.1038/srep26115)
Supplement: Supplementary Information [file srep26115-s1.doc]

**Supporting Information for**

**Multiple response optimization of the coagulation process for upgrading the quality of effluent from municipal wastewater treatment plant**

Na Li, Yi Hu, Yong-Ze Lu, Raymond J. Zeng, Guo-Ping Sheng^*^

CAS Key Laboratory for Urban Pollutant Conversion, Department of Chemistry, University of Science and Technology of China, Hefei, 230026, China

* **Corresponding author**:

Prof. Guo-Ping Sheng

Fax: 86-551-63601592

E-mail: gpsheng@ustc.edu.cn

Table S1

The main water quality indexes in the Surface Water Class Standard of China (GB3838-2002)

| **Class** |  | **Index (mg/L)** | | |  |
| --- | --- | --- | --- | --- | --- |
|  |  | NH_4_^+^-N | TP | COD | |
| **I**  II |  | 0.15  0.02 | 0.5  0.1 | 1.0  0.2 | |
| III |  | 15 | 15 | 20 | |
